# Clinicopathological Significance of PTEN Expression and Its Prognostic Effect in Colorectal Adenocarcinoma Patients

**DOI:** 10.30699/IJP.2021.531779.2653

**Published:** 2022-03-08

**Authors:** Zohreh Mirzapour Abbas Abadi, Fatemeh Samiee Rad, Dariush Hamedi Asl, Babak Rahmani, Mahmood Soleimani Dodaran, Amir Peimani

**Affiliations:** 1 Department of Molecular Medicine, Faculty of Medical Sciences, Qazvin University of Medical Sciences, Qazvin, Iran; 2 Department of Pathology, Faculty of Medical School, Qazvin University of Medical Sciences, Qazvin, Iran; 3 Department of Pathology and Molecular Medicine, Mehr Laboratory, Hashtgerd, Iran; 4 Medical Microbiology Research Center, Qazvin University of Medical Sciences, Qazvin, Iran

**Keywords:** Adenocarcinoma, Clinicopathology, Colorectal cancer, PTEN, Prognostic factors

## Abstract

**Background & Objective:**

Phosphatase and tensin homolog (PTEN) is a tumor suppressor gene located at chromosome 10. PTEN is a regulator of the PI3K/AKT signaling pathway that inhibits cell proliferation and promotes apoptosis. PTEN loss of function occurs in a spectrum of cancers, including colorectal adenocarcinoma. This study aimed to investigate the probable correlation of negative PTEN expression with clinicopathological features and colorectal adenocarcinoma (CRC) patients' survival.

**Methods:**

In this cross-sectional study using Immunohistochemistry stainingPTEN expression status on 151 CRC tissues was evaluated. Then the results of IHC staining was compared to those of clinicopathological features. The relationship between PTEN and KRAS mutation status was also investigated.

**Results:**

Of 151 CRC samples, 89 (58.9%) were negative for PTEN expression. Loss of PTEN expression was associated with KRAS mutation (*P*<0.0001), lymph node metastasis (*P*=0.002), and advanced tumor stage (*P*=0.016), whereas no significant association was found with other clinicopathological features. Multivariate analysis indicated that tumor site and KRAS mutation were independent prognostic CRC patients (*P*<0.05). The Kaplan-Meier analysis indicated a correlation between loss of PTEN expression and overall survival of patients with colorectal adenocarcinoma (*P*= 0.01).

**Conclusion:**

The current study suggests that decreasing PTEN expression or its negative expression may be associated with a higher stage and poor prognosis. Combined analysis of mutated KRAS and PTEN expression could be a good predictor of disease prognosis as well as its clinical outcomes.

## Introduction

Colorectal cancer (CRC) is the third common cancer (10% of all cancers in 2020) and the second cause of cancer-induced mortality worldwide (1). In colorectal adenocarcinoma, as with other types of cancer, several processes of genetic and epigenetic changes occur whereby normal endothelial cells are changed into cancer cells (2). The most critical signaling pathways associated with the epidermal growth factor receptor (EGFR) are RAS/RAF/MAPK and PIK3CA/PTEN-/AKT pathways, wherein colorectal adenocarcinoma the oncogenes and tumor suppressor genes of these pathways are mutated (3).

PI3K/AKT signaling pathway is involved in various cellular processes such as cell replication, apoptosis, and invasion (4). PTEN, located at human chromosome 10q23, is one of the essential negative regulators in the PI3K/AKT signaling pathway. By dephosphorylation of PI3K, this protein negatively regulates the AKT signaling pathway. Thus, PTEN guides the cell towards apoptosis and inhibits growth (5). In addition, PTEN participates in other cellular processes such as cell migration, cell cycle regulation, and tumor progression. Further, nuclear PTEN plays a crucial role in maintaining the stability of genetic information and regulates the Rad51 protein, which is one of the main components of DNA repair complexes (6).

PTEN loss of function can occur in a broad spectrum of cancers. This loss of function can occur through several molecular mechanisms. These mechanisms include mutation, deletion, and hyper promoter methylation that eventually causes loss of function of both alleles of this gene (7). PTEN inactivation is found in the vast majority of cancer, including glioblastoma, lung, gastric, breast, and other types of cancer. Similar observations have also been reported in colorectal adenocarcinoma, in which the PTEN expression gene has been suppressed (8, 9). A study reported that lack of expression of PTEN is associated with liver metastasis and local recurrence (10). In addition, other studies have shown that the status of PTEN expression predicts response to anti-EGFR monoclonal antibodies therapy (11, 12).

The RAS family includes some small GTPases (hydrolase enzymes that bind to GTP and hydrolyze it) that are major components of signal networks involved in cellular processes. Common molecular mutations occur in members of the RAS family (KRAS, NRAS, and HRAS) that cause tumor progression (13). *KRAS* gene mutations occur in 40-50% of CRC samples and are the most common mutations in this cancer. In contrast, *NRAS* and *HRAS* mutations occur with less frequency (1-3%). In 80% of cases, mutations occur in codons 12 and 13 of exon 2 of the *KRAS* gene (14). Studies have shown that mutations in the *KRAS* gene, in addition to causing resistance to targeted therapies with cetuximab and panitumumab, also have a significant effect on the prognosis of the disease (15, 16).

Various studies have shown an association between *PTEN* gene and clinicopathological characteristics in CRC patients. For example, loss of *PTEN* expression has demonstrated significant associations with lymph node metastasis, liver metastasis, and advanced TNM stage (10). Meanwhile, reducing *PTEN* expression is associ-ated with tumor size, lymphatic invasion, higher Dukes staging, and invasion depth (17, 18). Furthermore, the role of the PTEN gene as a prognostic marker has still not been proven. Nevertheless, various studies have shown the relationship between PTEN and survival of patients and poor clinical outcome (9, 10, 19). All these suggest that regulation of PTEN expression can be a good target for pharmacological interventions in CRC treatment (20). In this study, the expression of PTEN protein was examined using the immunohistochemistry technique in samples of colorectal adenocarcinoma patients. Then, the relationship between lack of express-ion of this protein and mutation of *KRAS* gene and clinic-pathological characteristics of patients was examined. It was also explored whether lack of expression of PTEN would affect the prognosis of patients.

## Material and Methods

In this cross-sectional study, a total of 151 formalin-fixed paraffin-embedded (FFPE) colorectal adenocarcinoma samples related to CRC patients who had undergone surgery between 2015 and 2018 were collected. Sampling was performed with a simple method. All samples were subjected to a checklist that included demographic information as well as clinical and histopathologic findings. The inclusion criteria included colorectal adenocarcinoma and sufficient pathological details. The exclusion criteria were insufficient pathological information. The clinical and pathological features of patients were evaluated. Stratification factors included: age, gender, site of a tumor, tumor size, pathological tumor stage, tumor differentiation, vascular invasion, lymphatic invasion, perineural invasion, lymph node metastasis, TNM stage. Hematoxylin and Eosin slides were reviewed by two pathologists. Survival time was considered from the day of diagnosing the disease until death or the last day of follow-up. The mean follow-up time was 33 months (range from 6 to 72 months).

I**mmunohistochemistry (IHC)**

In order to investigate the status of *PTEN* gene expression, two FFPE blocks were selected from each CRC patient, and 4 µm thick sections were mounted on poly l-lysine slides. First, the slides were depara-ffinized. For this purpose, the slides were exposed to 60°C for 15 min, and then deparaffinated for 5 min three times in xylene. The tissues were then rehydrated through exposure in alcohols 70, 90, and 100% and distilled water for 5 min. Antigen retrieval was perfor-med using sodium citrate buffer at pH=6 for 20 min at 97°C. After cooling, blocking of endogenous peroxi-dase activity was performed using peroxidase 0.3% solution for 15 min. After twice washing in phosphate-buffered saline (PBS) to prevent back-ground staining, blocking solution (POLHRP-006, Zytomed) was used for 15 min at room temperature. After three times of washing for 5 min in PBS solution, the tissues were incubated with PTEN antibody (clone: A2B1, code: sc-7974, Santacruz) for 1 h at room temperature. After washing with PBS, incubation with secondary antibody and DAB staining were performed according to the ZytoChem Plus HRP Polymer Kit (POLHRP-006, Zytomed) instructions. After counter staining using hematoxylin dye, tissue dehydration was performed according to the standard procedure. The specimens in which incubation with primary antibody has been eliminated were considered as negative control.

**Interpreting** ***PTEN*** **Expression**

**Fig. 1:**
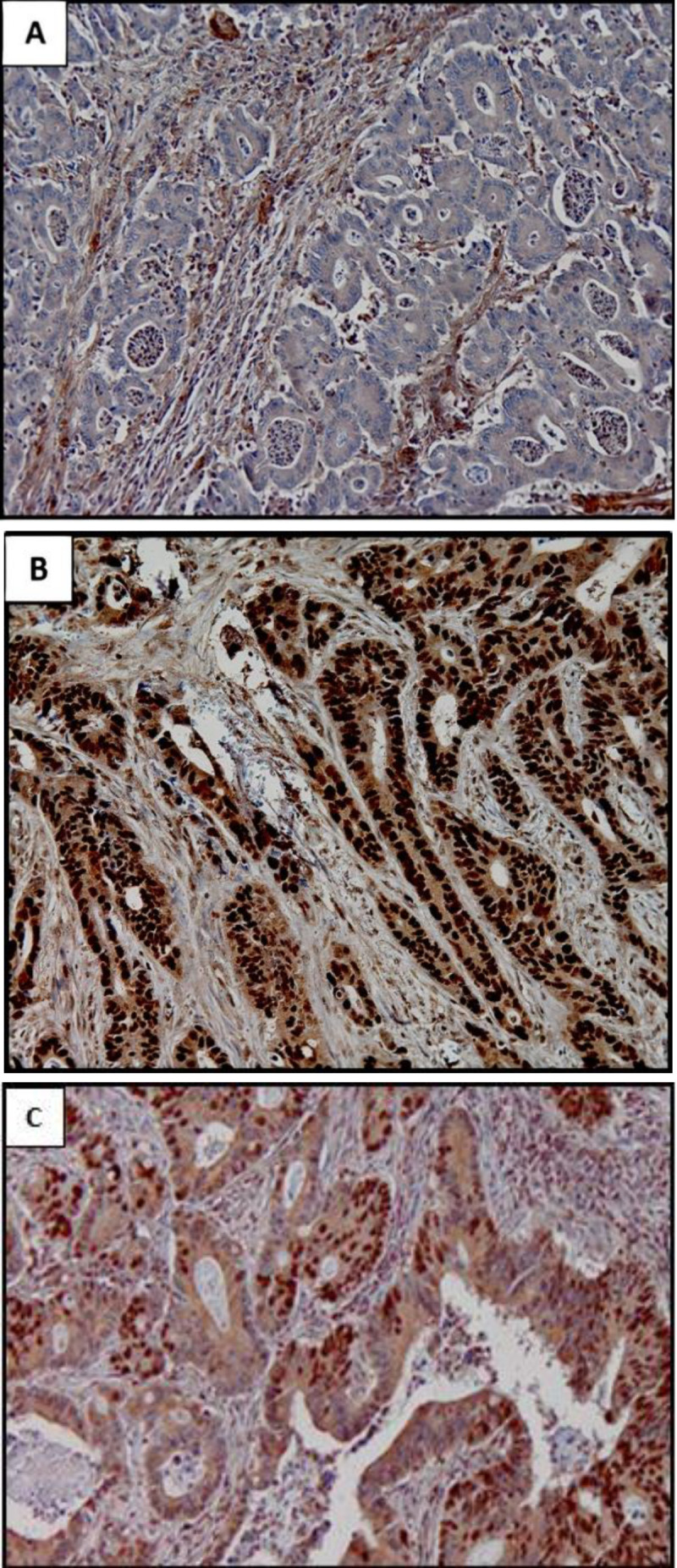
IHC staining results for PTEN in CRC tissues. A) Negative PTEN expression in cancer cells with positive expression in stromal and inflammatory cells. B) Positive PTEN expression in the cells of an adenocarcinoma

Two experienced pathologists studied the slides for patterns and intensities of immune staining in a blinded fashion. The results were similar in more than 97% of the cases. The rest of the cases were re-examined, and one single interpretation was presented. The stromal and inflame-matory cells observed in each slide were considered as positive internal control and scored +2 then the staining intensity of cancer cells was compared against them. The staining intensity was scored as +2 when equal to the positive control, +1 when poor or diminished staining compared to internal control, and negative when no immunostaining was observed. Immunos-taining with intensity greater than positive control cells was scored as +3. Considering the cancer cell heterogeneity, the cases in which more than 10% of cancer cells had staining with any intensity were considered positive (Figure 1).

**Statistical Analysis**

The relationship between expression of *PTEN* gene and clinicopathological characteristics of the patients included gender, site of tumor, histopathological subtype (adenocarcinoma, mucinous carcinoma), pathological tumor stage (primary tumor, extent of invasion), tumor differentiation, lymphatic invasion, vascular invasion, perineural invasion, number of lymph nodes metastasis, TNM stage, as well as the status of mutation of *KRAS* gene were detected through Chi-square test. Mann-Whitney U test was employed to compare the mean age and size of the tumor. In order to calculate overall survival, the Kaplan Meier method (using the Log Rank test) was used. Statistical analysis was carried out using SPSS 20 (SPSS Inc., Chicago, Ill., US), where P-value>0.05 was considered statistically significant**.**

## Results

**PTEN Expression Status and Clinicopatholo-gical Features**

All 151 tissue samples of colorectal adenocar-cinoma were examined for PTEN expression using the IHC test. *PTEN* expression has revealed nuclear expression in the cancer cell. In 89 (58.9%) CRC samples, *PTEN* showed negative expression (or had expression less than 10%), while 62 samples had positive expression. In the number of samples, heteroexpression of PTEN was observed, in which *PTEN* had expression in some cancer cells while others had no expression. These cases were considered positive.

The associations between *PTEN* expression and clinicopathological characteristics are shown in Table 1. Loss of *PTEN* expression was associated with lymph node metastasis (*P*=0.02) and higher stages (*P*=0.016). Nevertheless, no other relationship was found between expression of *PTEN* and gender, age, size of tumor, tumor site, Signet ring cell, mucinous component, pT stage, differentiation, lymphovascular invasion, perineural invasion, and angiovascular invasion.

**The Pattern of PTEN Expression and** ***KRAS*** **Status**

**Fig 2:**
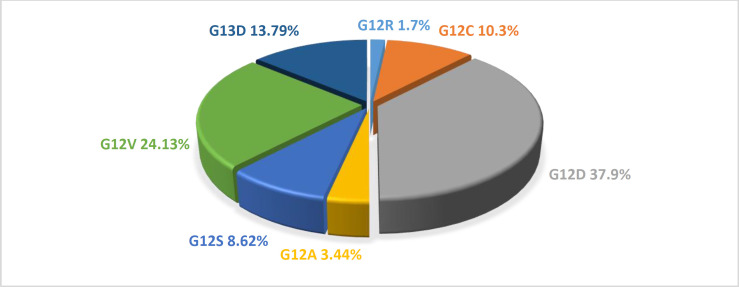
Frequency of KRAS mutations type . The most frequent mutation was G12D following by G12D

*KRAS* mutations of these 151 tumors samples have been evaluated using Pyrosequencing, and the method and results have been reported in our previous study (21). Out of 151 samples, 58 colorectal adenocarci-noma tissues had a mutation in one of the 12 or 13 codons. The distribution of *KRAS* mutations is shown in Figure 2. Loss of *PTEN* gene expression was significantly associated with mutated *KRAS* (Table 1). The mutated *KRAS* was not only particularly associated with negative *PTEN* but was also related to the intens-ity of *PTEN* expression; The *PTEN* expression was down-regulated in tumors with *KRAS* mutated status (Table 2). Further, the tumors carrying *KRAS* in codon 13 were associated with negative PTEN (Table 3).

**Table 1:** Correlation between PTEN expression status and clinicopathological parameters in 151 colorectal adenocarcinomas

Clinicopathologic Feature	All (%.n=153)	PTEN expression Negative(%) Positive(%)		P-value (χ^2^ test)
Age <60 ⩾60	73 (48.3) 78 (51.7)	33 (53.2) 29 (46.8)	40 (44.9) 49 (55.1)	**0.2**
Sex Female Male	61(40.4) 90(59.6)	27(43.5) 35(56.5)	34(38.2) 55(61.8)	**0.31**
Tumor site Proximal colon Distal colon Rectum	51(33.8) 78(41.7) 22(14.6)	23 (37.1) 27 (43.5) 12 (19.4)	38(31.5) 50(57.3) 10(11.2)	**0.18**
Size of tumor (cm) <5 ⩾5	53 (35.1) 98 (64.1)	16 (25.8) 46 (74.2)	37 (41.6) 52 (58.4)	**0.056**
Adenocarcinoma, NOS Without mucinous component With mucinous component	119(78.8) 32(21.2)	46(74.2) 16(25.8)	73(82) 16(18)	**0.16**
pT stage PT1-2 PT3-4	27(17.9) 124(82.1)	9(14.5) 53(85.5)	18(20.2) 71(79.8)	**0.36**
Differentiation Well Moderate Poor	89(58.9) 52(34.4) 5(3.3)	32(51.6) 25(40.3) 3(4.8)	57(51.6) 27(40.3) 2(2.2)	**0** **.** **43**
pT stage PT1-2 PT3-4	27(17.9) 124 (82.1)	9 (14.5) 53 (85.5)	18(20.2) 71(79.8)	**0.36**
Signet ring cell >50% <50% Absent	0 6(4) 145(96)	0 4(6.5) 58 (93.5)	0 2(2.2) 87(97.8)	**0.19**
Mucinous component Absent Present	119(78.8) 32(21.2)	46(74.2) 16(25.8)	73(82) 16(18)	**0.16**
Lymphovascular invasion Absent Present	123(81) 28(19)	52(83.9) 10(16.1)	71(79.8) 18 (20.2)	**0.33**
Angiovascular invasion Absent Present	121 (80) 30 (20)	53 (85.5) 9(14.5)	68 (76.4) 21 (23.6)	**0.16**
Perineural invasion Absent Present	124 (82.1) 27 (17.9)	52 (83.9) 10 (16.1)	72 (80.9) 17 (19.1)	**0.17**
pN stage PN0 PN1-2	110 (72.8) 41 (27.2)	37 (59.7) 25 (40.3)	73 (82) 16 (18)	**0.002**
TNM stage I-II III-IV	95 (62.9) 56 (37.1)	32 (51.6) 30 (48.4)	63 (70.8) 26 (29.2)	**0.016**
KRAS status Mutated Wild	**58 (38.4)** **93 (61.6)**	**39(62.9)** **23(37** **.** **1)**	**19(21.3)** **70(78** **.** **7)**	**<0.0001**

**Table 2:** Association between different PTEN expression and KRAS status

	All N (%)	PTEN expression				P-value
		0	1+	2+	3+	
Mutated KRAS status	58 (38.4)	39 (62.9)	9 (30)	9 (20.9)	1 (6.2)	**<0.0001**
Wild-Type	**93** **(61.6)**	**23** **(37.1)**	**21** **(70)**	**34** **(79.1)**	**15** **(93.8)**

**Table 3:** Association between PTEN expression status and the location of KRAS mutations

		KRAS status			All N (%)	P -value
		Wild-type	codon 12 mutated	codon 13 mutated		
PTEN	**Negative**	23(24.7)	33(66)	6(75)	62(41.1)	**<0.0001**
status	Positive	**70(75.3)**	**17(34)**	**2(25)**	**89(58.9)**

**PTEN Expression Status and Overall Survival**

**Fig. 3:**
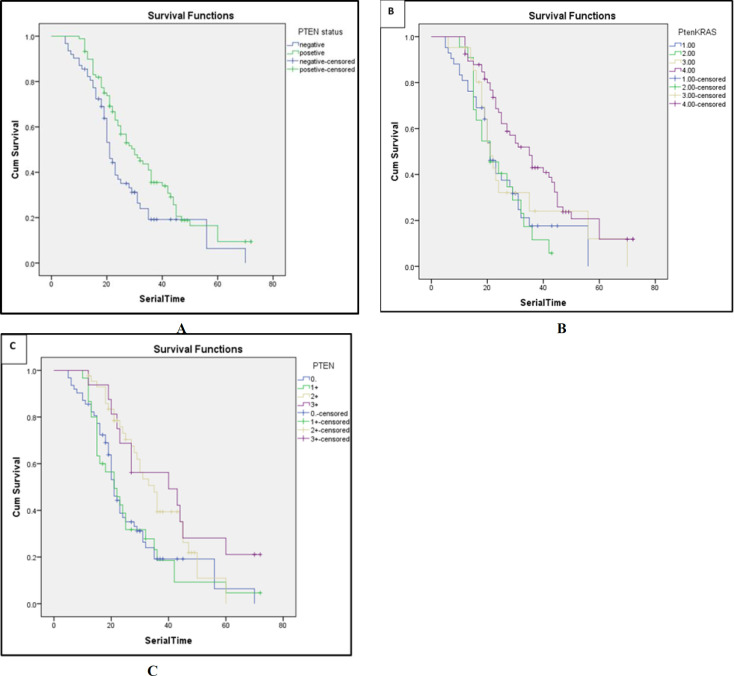
**A)** Survival curves for all patients with positive and negative PTEN expression. **B)** survival curves with regarding to PTEN expression and KRAS mutation status in all cases (1. PTEN negative+KRAS mutated 2. PTEN Posetive+KRAS mutated 3. PTEN negative+KRAS wild type 4. All wild type). **C)** Survival curves in different intensity of PTEN expression in all cases

Kaplan-Meier analysis in stage I-IV indicated decreased overall survival in PTEN negative tumors compared to PTEN positive tumors (*P*=0.01) (Figure 3A). Investigation of overall survival at different IHC scores showed that OS has decreased following the reduction of PTEN expression protein (*P*=0.004) (Figure 3C). Concurrent analysis of *KRAS* and *PTEN* mutation showed that mutation in *KRAS* gene and negative *PTEN* had a considerable impact on survival of patients compared to patients who had wild type in both genes (*P*=0.004) (Figure 3B).

Cox regression analysis was performed to investigate the prognostic value of clinicopathological characteristics and gene mutations. In multivariate analysis, the mutation in the *KRAS* gene was significa-ntly associated with worse survival (*P*=0.008). How-ever, this association wasn't observed with negative *PTEN* (*P*=0.4). Regarding other clinicopathological characteristics, only the tumor site was considered as an independent prognostic factor (*P*=0.01) (Table 4).

**Table 4:** Multivariate analysis of clinicopathologic features

Prognosis variables	Stage I-IV	
	P-value	**HR(95% CI)**
Sex (female)	0.48	**1.15(0.77-1.70)**
Age	0.12	**1.31(0.89-1.9)**
Differentiation	0.53	**1.2(0.67-2.14)**
Tumor size	0.4	**1.2(0.78-1.85)**
Tumor site	0.01	**0.6 (0.4-0.89)**
Perineural invasion	0.55	**1.16(0.7-1.91)**
Angiovascular invasion	0.77	**1.06(0.68-1.64)**
KRAS mutant	0.008	**1.72(1.15-2.57)**
PTEN (negative)	**0.4**	**1** **.** **17(0.81-1.68)**

## Discussion

Detecting biomarkers that affect patients’ clinical outcomes is a critical step for choosing a treatment approach with maximum efficiency. Indeed, research on targeted therapies aims to identify the markers that affect disease prognosis or cause resistance to a particular type of treatment (22). *PTEN* is a tumor suppressor gene, which is involved in various cellular processes, including survival, proliferation, cellular metabolism, genome stability, differentiation, and apoptosis (23, 24). The mutations and mechanisms that lead to the failure of PTEN expression play a vital role in the development and metastasis of CRC tumors (25). In various studies, the PTEN loss of expression rate is significantly different (5.8-50%) (8, 9, 26). In this study, IHC results showed negative expression of PTEN in 58.9% (89/151) of tumors. The negative *PTEN* level in other studies on the Iranian population was slightly lower compared to this study (19).

Various studies have shown that the status of *PTEN* expression can affect patients' clinical and pathological characteristics. Colakoglu *et al.* compared the expression of *PTEN* in CRC patients with clinicopath-ological characteristics. They found a negative correlation between expression of PTEN and younger age, female sex, and left-sided tumors (8). On the other hand, other studies showed no association between clinicopathological characteristics and negative expression of the *PTEN* gene (27, 28). Our study showed that loss of *PTEN* expression was related to lymph node metastasis and advanced tumor stage. Since negative changes of *PTEN* expression would cause cell proliferation, inhibited apoptosis, and increased tumor aggression (5, 29), the relationship between negative *PTEN* and metastasis as well as tumor progression (high stage) is interpretable. Comp-arison of the *KRAS* gene status with *PTEN* expression in this study showed a strong relationship between mutated *KRAS* and negative *PTEN* expression. Further analyses showed that reduced expression of PTEN in tumors is also associated with mutated KRAS. We found no significant relationship between PTEN expression status and age, tumor size, gender, tumor site, Signet ring cell, mucinous component, pT stage, differentiation, and vascular invasion.

Although the role of the *PTEN* gene as a prognostic marker is still controversial; various studies have shown that decreased expression of this protein in CRC tumors is associated with lymph node and liver metast-asis and can cause lower patient survival (9, 10). Another study stated that during the development, aggression, and metastasis of tumors, the *PTEN* gene promoter becomes methylated, leading to decreased *PTEN* expression (30). Another study indicated that during the growth and invasion of CRC tumor, *PTEN* gene promoter is methylated and inactivated, event-ually causing decreased *PTEN* expression (19). In this study, the OS of patients was examined based on the status of *KRAS* and *PTEN*. In agreement with other studies (9, 10, 19), survival analysis in CRC patients indicated that loss of *PTEN* gene expression or reduced expression of *PTEN* could cause reduced survival.

Nevertheless, multivariate analysis showed , no considerable effect for negative *PTEN* on negative clinical outcomes in CRC patients. Furthermore, for the combined impact of *KRAS* mutation and *PTEN* expression on OS, the results showed that concurrent presence of two favorable prognostic factors compared to only one of the markers or absence of both markers would lead to better survival of patients. Thus, it can be concluded that concurrent analysis of mutated *KRAS* and *PTEN* expression in patients would be helpful for a better prediction of prognosis and clinical outcomes, although it requires further research with larger sample size.

## Conclusion

Our results suggested that decreased expression of *PTEN* or its loss of expression may be associated with tumor progression, and negative *PTEN* expression can be associated with a higher tumor stage and poor prognosis. Combined analysis of mutated *KRAS* and *PTEN* expression can be a good predictor of disease prognosis as well as its clinical outcomes. This highlights the necessity of investigation of these two markers in all CRC patients. Nevertheless, future research should be expanded with larger sample size to more clearly confirm these resultsr.

## Author Contributions

ZMA, DHA, BR, and AP designed and oversaw the study. ZMA and DHA performed the experiments. FSR and MSD contributed to data collection and analysis. ZMA and DHA drafted the manuscript and final approval of the version to be published. All authors read and approved the final manuscript. 

## Conflict of Interest

The authors declared no conflict of interest.

## Funding

This work was supported by Grant-in-Aid for Scientific Research of Qazvin University of Medical Sciences (NO.14003016).
